# Undiagnosed Diabetes in Metabolically Unhealthy Normal Weight Adults: A Cross-Sectional Analysis of National Health and Nutrition Examination Survey Cycle 2017–2020 in the United States

**DOI:** 10.3390/jcm15041385

**Published:** 2026-02-10

**Authors:** Sándor Pál, Annamária Sepsey

**Affiliations:** 1 Department of Transfusion Medicine, Department of Laboratory Medicine, University of Pécs Medical School, 7624 Pécs, Hungary; 2 Department of Bioanalysis, University of Pécs Medical School, 7624 Pécs, Hungary

**Keywords:** undiagnosed diabetes, metabolically unhealthy normal weight (MUNW), cross-sectional study, United States, NHANES, body mass index, clinical screening

## Abstract

**Background/Objectives**: Although body mass index (BMI) is a conventional screening tool for type 2 diabetes mellitus (T2D), its reliability as a sole indicator of metabolic health is controversial, and the metabolic profile of a subset of individuals with normal BMI is indicative of obesity-related complications. This study aimed to estimate the prevalence and predictors of undiagnosed diabetes among Metabolically Unhealthy Normal Weight (MUNW) adults. **Methods**: Data from the National Health and Nutrition Examination Survey (NHANES) 2017–March 2020 were analyzed. Normal weight adults (BMI 18.5–24.9 kg/m^2^) were categorized into Metabolically Healthy (MHNW) and Unhealthy (MUNW) phenotypes based on the presence of ≥2 metabolic risk factors, including elevated blood pressure, triglycerides, waist circumference, or low HDL cholesterol. The primary outcome was undiagnosed diabetes, defined as HbA1c ≥ 6.5% or Fasting Plasma Glucose ≥ 126 mg/dL. **Results**: The study population represented approximately 60 million US adults. The prevalence of undiagnosed diabetes was nearly four times higher in the MUNW group (4.84%) compared to the MHNW group (1.28%). In multivariable logistic regression analysis, age and race emerged as significant predictors. Notably, Asian adults exhibited a significantly higher risk of undiagnosed diabetes (OR 6.10; 95% CI: 1.32–28.2) compared to White adults, independent of metabolic phenotype. **Conclusions**: Reliance solely on BMI may overlook undiagnosed diabetes in normal-weight adults, particularly those with metabolic clustering or of Asian descent. These findings underscore the importance of multidimensional risk assessment integration into preventive care, optimizing clinical management.

## 1. Introduction

Type 2 Diabetes Mellitus (T2D) has attained epidemic status on a global scale, with the International Diabetes Federation reporting that over 537 million adults were affected by diabetes in 2021 [1]. This figure is anticipated to increase to 783 million by 2045 [2]. A significant issue within this epidemiological crisis is the phenomenon of the “missing millions”—individuals who remain undiagnosed with diabetes mellitus and, as a result, are untreated and at elevated risk for both microvascular and macrovascular complications [3].

The COVID-19 pandemic introduced significant complexity to chronic disease surveillance. During 2020–2022, disruptions in routine healthcare access, altered dietary behaviors, and decreased physical activity during lockdowns likely influenced metabolic health population-wide [4]. Furthermore, data collection for major surveillance systems, such as the National Health and Nutrition Examination Survey (NHANES), was suspended or methodologically altered during the height of the pandemic. Consequently, prevalence estimates derived from the mid-pandemic period may reflect transient environmental stressors or selection biases due to limited healthcare utilization. Establishing a robust baseline using pre-pandemic data (up to March 2020) is therefore essential to accurately understand the structural prevalence of undiagnosed diabetes before the systemic disruptions of COVID-19.

The pathophysiology of T2D is classically linked to excess adiposity, with Body Mass Index (BMI) serving as the primary clinical surrogate for risk. However, BMI is a measure of excess weight, not excess body fat or distribution. A distinct sub-population exists that possesses a normal BMI (18.5–24.9 kg/m^2^) but exhibits a clustering of metabolic abnormalities typically associated with obesity—a condition termed “Metabolically Unhealthy Normal Weight” (MUNW) [3,5,6]. In a modern, multidimensiona and multidisciplinary organizational context, reliance on BMI alone fails to address the complexities of patient management and preventive care, where accurate risk stratification is vital for early intervention [7,8,9].

“Metabolically unhealthy” in this context refers to a state of insulin resistance characterized by specific biomarkers, including dyslipidemia (high triglycerides, low HDL-C), hypertension, and central adiposity (increased visceral fat deposits despite normal overall weight) [6,10].

Mechanisms proposed for the MUNW phenotype include genetic susceptibility, low birth weight, and sarcopenia (low muscle mass), which limits glucose disposal capacity [11,12,13,14].

Since screening guidelines often prioritize individuals who are overweight or obese (BMI ≥ 25 kg/m^2^) [15], those with a metabolically unhealthy normal weight (MUNW) might be overlooked in primary care surveillance [4]. The primary objectives were to estimate the prevalence of undiagnosed diabetes specifically among normal-weight US adults, categorized by metabolic health, while the secondary objectives were to identify sociodemographic predictors that could assist clinicians in recocnizing high-rik normal-weight patients.

## 2. Materials and Methods

### 2.1. Data Source and Study Population

We utilized data from the NHANES 2017–March 2020 cycle (P_dataset) [4]. NHANES is a cross-sectional survey conducted by the National Center for Health Statistics (NCHS) using a complex, multistage probability design to represent the civilian, non-institutionalized US population. This specific cycle combines data from 2017–2018 and the partial 2019–2020 cycle, which ended in March 2020 due to the COVID-19 pandemic. This study was conductd in accordance with the Strengthening the Reporting of Observational Studies in Epidemiology (STROBE) guidelines [16]. A completed STROBE checklist is provided in the Supplementary Materials.

**Figure 1:**
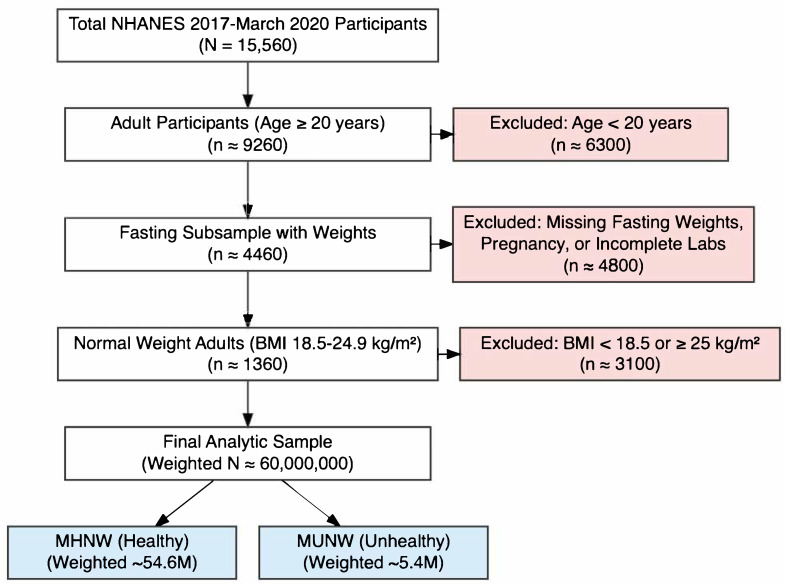
Case–selection flow–diagram. Legend: NHANES = National Health And Nutrition Examination Survey; BMI = Body Mass Index; MHNW = Metabolically Healthy Normal Weight; MUNW = Metabolically Unhealthy Normal Weight.

Participants were included if they aged at least 20 years, classified as “Normal Weight” (BMI ≥ 18.5 and <25 kg/m^2^) and had complete data for diabetes diagnosis questions, HbA1c, fasting plasma glucose, and components of metabolic syndrome. Exclusion criteria contained missing weights for the fasting subsample, pregnancy at the time of the examination and incomplete data regarding the primary outcome ariables defined below. The case selection flow-diagram is represented in Figure 1.

### 2.2. Definition of Metabolic Phenotypes

Metabolic health was defined using a harmonized definition derived from the NCEP ATP III criteria. Individuals were classified as Metabolically Unhealthy Normal Weight (MUNW) if they possessed two or more of the following risk factors (excluding hyperglycemia, which was the study outcome): elevated triglycerides (≥150 mg/dL), reduced HDL cholesterol (<40 mg/dL for men or <50 mg/dL for women), elevated blood pressure (systolic ≥ 130 mmHg OR Diastolic ≥ 85 mmHg) and presented central adiposity (Waist Circumference) (>102 cm for men or >88 cm for women). The cutoff of ≥2 abnormalities was selected to balance sensitivity and specificity while excluding the outcome variable prevent false statistical results. Individuals with 0 or 1 risk factor were classified as Metabolically Healthy Normal Weight (MHNW).

### 2.3. Defining Diabetes Status

The determination of diabetes status was conducted through a sequential algorithm. Individuals were classified as having diagnosed diabetes if they responded affirmatively to the query, “Have you ever been told by a doctor or health professional that you have diabetes?” These individuals were subsequently excluded from the analysis of “undiagnosed” diabetes (or coded as 0, contingent upon the specific model target). Undiagnosed diabetes encompassed participants who reported no prior diagnosis but met clinical criteria for diabetes, such as Hemoglobin A1c (HbA1c) ≥ 6.5% or Fasting Plasma Glucose (FPG) ≥ 126 mg/dL. Discordant cases (elevated HbA1c with normal FPG) were classified as undiagnosed diabetes capturing the full spectrum of glycemia-related disorders.

### 2.4. Statistical Analysis

All statistical analyses accounted for the complex survey design of NHANES. The fasting subsample weights (WTSAFPRP) were used to account for the subset of participants who underwent fasting blood draws (glucose/triglycerides). Variance estimation used the Taylor series linearization method (also known as delta method) via the masked variance units (SDMVPSU and SDMVSTRA). Baseline characteristics between MHNW and MUNW groups using design-adjusted Chi-square tests (Rao & Scott adjustment) were performed. Weighted prevalence estimates with 95% Confidence Intervals (CI) were calculated for undiagnosed diabetes within each phenotype. A survey-weighted multivariable logistic regression model (quasibinomial family) was constructed to assess the association between phenotype and undiagnosed diabetes, adjusting for potential confounders: Age (20–39, 40–59, 60+), Gender, Race/Ethnicity, Family History of Diabetes, Smoking Status, Income to Poverty Ratio (PIR), and Education level.

All analyses were performed using R statistical software (R Core Team, Vienna, Austria) (version 4.5.1) (using survey, tidyverse, and gtsummary packages). Statistical significance was set at *p* < 0.05.

## 3. Results

The weighted analytic sample represented approximately 60 million normal-weight US adults. Within this population, the majority (approx. 54.5 million) were classified as Metabolically Healthy (MHNW), while a significant subset (approx. 5.3 million) met the criteria for Metabolically Unhealthy (MUNW).

As detailed in Table 1, the MUNW population differed significantly from the healthy group in age and education.

The MUNW group was significantly older; 49% of the MUNW group was aged 60+, compared to only 22% in the MHNW group. Educational attainment also differed significantly, with a lower proportion of college graduates in the MUNW group (51%) compared to the MHNW group (68%).

The prevalence of undiagnosed diabetes varied starkly by metabolic health status. The prevalence of undiagnosed diabetes in the MHNW group was 1.28% (95% CI: −0.01% to 2.57%). We found a diabetes prevalence of 4.84% (95% CI: 1.20% to 8.47%) in the MUNW group, therefore, normal-weight adults with metabolic clustering were nearly four times more likely to harbor undiagnosed diabetes than their healthy counterparts.

In the fully adjusted logistic regression model (Table 2), we examined independent predictors of undiagnosed diabetes.

The MUNW phenotype showed an Odds Ratio (OR) of 2.35 (95% CI: 0.57–9.68). While clinically substantial, this did not reach statistical significance (*p* = 0.2), likely due to the strong confounding effect of age and wide confidence intervals inherent in the small sub-population of undiagnosed cases.

Age was the most powerful predictor. Compared to adults aged 20–39, those aged 40–59 had an OR of 20.9 (*p* = 0.010), and those aged 60+ had an OR of 11.8 (*p* = 0.024).

Non-Hispanic Asian adults exhibited a markedly elevated risk. Compared to Non-Hispanic White adults, Asian adults had an OR of 6.10 (95% CI: 1.32–28.2, *p* = 0.026). Hwever, the wide confidence intervals observed in ethnicity-specific analyses indicate limited statistical power in certain subgroups.

Individuals with “Some College” education had higher odds (OR 4.28) compared to college graduates (*p* = 0.039).

## 4. Discussion

This study identifies a critical gap in diabetes surveillance: nearly 5% of normal-weight adults with metabolic abnormalities have undiagnosed diabetes. While 5% may appear low compared to obese populations, applied to the US population, this represents hundreds of thousands of individuals who are unlikely to be screened under traditional BMI-centric protocols. The contrast with the 1.28% prevalence in the healthy phenotype confirms that BMI is an insufficient filter for metabolic risk assessment.

Our results highlight that “normal weight” has different meaning when considering patient demographics. In this study, the strongest driver of undiagnosed diabetes was age. This suggests that in older adults, BMI becomes a less reliable indicator of metabolic health, likely due to the age-related redistribution of fat from subcutaneous to visceral depots and due to continuous loss of muscle mass (sarcopenia) [17,18]. These phenomena are also accompanied by leptin and insulin resistance and many other metabolic changes, leading to cardiometabolic dysfunctions and increased risk of morbidity and mortality [18,19].

The strikingly high Odds Ratio (OR: 6.10) observed for Asian adults in this study reinforces the concept that this population generally has a lower personal fat threshold. Asian individuals may develop insulin resistance and T2D at much lower BMI levels than Caucasians. Due to ethnic differences in body composition, fat distribution, inflammation, and genetic factors, Asian populations develop insulin resistance and T2D at lower BMI levels than Caucasians. This necessitates ethnicity-specific BMI cutoffs and earlier metabolic screening in Asians to enable timely prevention and intervention for diabetes and related metabolic diseases [20,21,22,23,24,25]. The American Diabetes Association’s recommendation to screen Asian Americans at a BMI of ≥23 kg/m^2^, rather than the standard ≥ 25 kg/m^2^, is also supported by these findings [26]. Current USPSTF (United States Preventive Services Task Force) guidelines recommend screening for prediabetes and diabetes in adults aged 35 to 70 years who have overweight or obesity [27]. During clinical practice a transition from a purely weight-centric screening model to one that incorporates metabolic health and patient history, particularly in preventive care settings would enable a much more efficient diabetes mellitus screening.

Thus, in a modern healthcare environment, the management of patients with MUNW requires a multidisciplinary approach. Implementing standardized care pathways and clinical networks can help achieve quadruple aim goals–improving patient experience, population health, and provider well-being while reducing costs–by ensuring that these high-risk individuals are not overlooked [7,8,9].

Clinicians should assess metabolic changes beyond BMI to detect metabolically unhealthy normal-weight (MUNW) adults during check-ups. Key indicators include hypertriglyceridemia (≥150 mg/dL) and elevated blood pressure (≥130/85 mmHg). These findings should prompt HbA1c testing to identify dysglycemia risk. Waist circumference is crucial for detecting abnormal fat distribution in MUNW individuals, as it signifies visceral adiposity associated with metabolic abnormalities despite normal BMI. Evidence-based recommendations suggest that in the absence of elevated BMI, the presence of at least two components of metabolic syndrome should trigger screening for diabetes mellitus.

Bioelectrical impedance shows increased adiposity and decreased muscle mass link to unfavorable metabolic traits in normal-weight adults. Molecular and genetic markers differ between metabolically healthy and unhealthy phenotypes independent of BMI. Altered expression of microRNAs, inflammatory markers, and pathways in lipid and glucose metabolism characterize MUNW individuals. Genetic studies have identified loci associated with metabolically unhealthy phenotypes in normal-weight subjects. The cardiometabolic index (CMI) shows promising accuracy in identifying MUNW individuals, outperforming BMI alone. Detecting MUNW adults requires assessment of metabolic profiles, including lipids, blood pressure, HbA1c, and waist circumference, enabling early intervention for at-risk individuals [28,29,30,31,32]. The high OR in the 40–59 age group supports lowering the universal screening age to 35 (as recently updated by the ADA), regardless of weight, to also capture these cases, still receiving less attention [33].

For the MUNW population, standard advices aiming body mass reduction may be counterproductive or confusing, as they are already within the normal weight range. Interventions should focus on body composition rather than weight loss. Resistance training is crucial to combat sarcopenia and improve insulin sensitivity in skeletal muscle [34,35,36]. Emphasis should shift to reducing refined carbohydrates and saturated fats that contribute to visceral fat deposition, even in the absence of caloric surplus.

### Strengths and Limitations

Strengths of this study include the use of the high-quality, pre-pandemic NHANES dataset, which provides a clean baseline unaffected by COVID-19 disruptions. However, the reliance on BMI as a primary selection criterion for “normal weight” inherently carries limitations, as it does not differentiate between lean mass and fat mass, potentially misclassifying individuals with sarcopenic obesity, but may also include individuals with increased muscle mass into the group with high BMI. Furthermore, the relatively small sample size of undiagnosed cases within the normal-weight strata resulted in wide confidence intervals, limiting the statistical power to detect significant differences in smaller subgroups, and restricting generalizability. Finally, sensitivity analyses using alternative definitions of metabolic health were not conducted, which is also a limiting factor.

## 5. Conclusions

Normal weight is not a guarantee of healthy metabolic status. A specific subset of the population—older adults, those of Asian descent, and those with clustered metabolic risk factors—face a significantly elevated risk of undiagnosed diabetes despite having a normal BMI. Therefore, clinical practice should evolve from a weight-centric view to a lipocentric and metabolic view. Early identification of the MUNW phenotype through expanded screening criteria is essential to preventing the long-term complications of untreated diabetes in this overlooked population.

## Figures and Tables

**Table 1:** Weighted Characteristics of Normal Weight Adults (NHANES 2017–2020).

Characteristic	MHNW (Healthy)	MUNW (Unhealthy)	*p*-Value
**N (Weighted)**	~54,561,896	~5,344,767	
**Age Group**			**0.005**
20–39	49%	22%	
40–59	29%	29%	
60+	22%	49%	
**Gender**			0.5
Male	45%	39%	
Female	55%	61%	
**Race/Ethnicity**			0.2
White	66%	62%	
Black	9.0%	7.1%	
Asian	9.7%	16%	
Mexican American	6.4%	5.5%	
Other Hispanic	5.7%	3.7%	
Other	3.7%	5.8%	
**Education**			**0.005**
College Grad	68%	51%	
Some College	24%	27%	
HS or Less	7.8%	22%	
**Income**			0.5
High Income	54%	54%	
Middle Income	31%	35%	
Low Income	15%	11%	
**Smoking Status**			0.2
Never	60%	56%	
Former	24%	17%	
Current	16%	28%	
**Family History**	36%	49%	0.2

Note: Values are weighted percentages. Legend: MHNW: Metabolically Healthy Normal Weight; MUNW: Metabolically Unhealthy Normal Weight; HS: High School; N: Estimated population count based on survey weights.

**Table 2:** Adjusted Odds Ratios for Undiagnosed Diabetes Mellitus.

Characteristic	OR	95% CI	*p*-Value
**Phenotype**			
MHNW (Healthy)	Ref.	—	—
MUNW (Unhealthy)	2.35	0.57–9.68	0.2
**Age Group**			
20–39	Ref.	—	—
40–59	20.9	2.57–170	**0.010**
60+	11.8	1.49–92.5	**0.024**
**Race/Ethnicity**			
White	Ref.	—	—
Asian	6.10	1.32–28.2	**0.026**
Black	3.24	0.55–19.0	0.2
Other Hispanic	7.53	0.36–156	0.2
Other	0.35	0.01–18.3	0.6
Mexican American	0.00	0.0–0.00	**<0.001**
**Education**			
College Grad	Ref.	—	—
Some College	4.28	1.10–16.7	**0.039**
HS or Less	1.52	0.32–7.07	0.6
**Other Covariates**			
Gender (Female Ref: Male	2.04	0.34–12.1	0.4
Family Hx (Yes Ref: No)	2.19	0.45–10.6	0.3
Smoking (Current)	Ref.	—	—
Smoking (Former)	0.24	0.03–2.00	0.2
Smoking (Never)	0.25	0.03–1.78	0.14

Note: Model adjusted for all variables listed. Other covariates’ reference group is either presented instead of OR values, or presented in brackets. Legend: OR: Adjusted Odds Ratio; CI: Confidence Interval; MHNW: Metabolically Healthy Normal Weight; MUNW: Metabolically Unhealthy Normal Weight; Ref: Reference group. The model is adjusted for age, gender, race, education, family history, and smoking status.

## Data Availability

The data in this manuscript is reused from the following source link: https://wwwn.cdc.gov/nchs/nhanes/continuousnhanes/default.aspx?Cycle=2017–2020, last accessed on 4 December 2025.

## Supplementary Materials

The following supporting information can be downloaded at: https://www.mdpi.com/article/10.3390/jcm15041385/s1, Table S1: STROBE statement.

## Abbreviations

The following abbreviations are used in this manuscript:

ADA	American Diabetes Association
BMI	Body Mass Index
CI	Confidence Interval
CMI	Cardiometabolic Index
COVID-19	Coronavirus Disease 2019
FPG	Fasting Plasma Glucose
HbA1c	Hemoglobin A1c
HDL	High-Density Lipoprotein
HDL-C	High-Density Lipoprotein Cholesterol
MHNW	Metabolically Healthy Normal Weight
MUNW	Metabolically Unhealthy Normal Weight
NCEP ATP III	National Cholesterol Education Program Adult Treatment Panel III
NCHS	National Center for Health Statistics
NHANES	National Health and Nutrition Examination Survey
OR	Odds Ratio
PIR	Poverty Income Ratio
T2D	Type 2 Diabetes Mellitus
USPSTF	United States Preventive Services Task Force
