# Supplementary material for: Undiagnosed Diabetes in Metabolically Unhealthy Normal Weight Adults: A Cross-Sectional Analysis of National Health and Nutrition Examination Survey Cycle 2017–2020 in the United States
Source: J Clin Med. 2026 Feb 10;15(4):1385. doi: 10.3390/jcm15041385 (PMC12942309; doi:10.3390/jcm15041385)
Supplement: Supplementary file 1 [file jcm-15-01385-s001.zip › jcm-4053560-supplementary.pdf]

STROBE Statement

SECTION/TOPIC	ITEM NO	RECOMMENDATION	PAGE/SECTION & SENTENCE START
**TITLE AND ABSTRACT**			
**TITLE**	1a	Indicate the study’s design with a commonly used term in the title or the abstract	**Title**<br><br>"Undiagnosed Diabetes in Metabolically Unhealthy..."
**ABSTRACT**	1b	Provide in the abstract an informative and balanced summary of what was done and what was found	**Abstract**<br><br>"Although body mass index (BMI) is a conventional..."
**INTRODUCTION**			
**BACKGROUND/RATIONALE**	2	Explain the scientific background and rationale for the investigation being reported	**1. Introduction**<br><br>"Type 2 Diabetes (T2D) has attained epidemic status..."
**OBJECTIVES**	3	State specific objectives, including any prespecified hypotheses	**1. Introduction**<br><br>"The primary objectives were to estimate the prevalence..."
**METHODS**			
**STUDY DESIGN**	4	Present key elements of study design early in the paper	**2.1. Data Source and Study Population**<br><br>"We utilized data from the NHANES..."
**SETTING**	5	Describe the setting, locations, and relevant dates, including periods of recruitment, exposure, follow-up, and data collection	**2.1. Data Source and Study Population**<br><br>"This specific cycle combines data from 2017–2018..."
**PARTICIPANTS**	6	(a) Give the eligibility criteria, and the sources and methods of selection of participants	**2.1. Data Source and Study Population**<br><br>"Participants were included if they aged at least..."
**VARIABLES**	7	Clearly define all outcomes, exposures, predictors, potential confounders, and effect modifiers. Give diagnostic criteria, if applicable	**2.2. Definition of Metabolic Phenotypes**<br><br>"Metabolic health was defined using a harmonized..."

**DATA SOURCES/ MEASUREMENT**	8*	For each variable of interest, give sources of data and details of methods of assessment (measurement). Describe comparability of assessment methods if there is more than one group	**2.1. Data Source and Study Population**<br><br>"We utilized data from the NHANES 2017–March..."
**BIAS**	9	Describe any efforts to address potential sources of bias	**2.4. Statistical Analysis**<br><br>"The fasting subsample weights (WTSAFPRP) were used..."
**STUDY SIZE**	10	Explain how the study size was arrived at	**3. Results**<br><br>"The weighted analytic sample represented approximately 60 million..."
**QUANTITATIVE VARIABLES**	11	Explain how quantitative variables were handled in the analyses. If applicable, describe which groupings were chosen and why	**2.2. Definition of Metabolic Phenotypes**<br><br>"The cutoff of ≥2 abnormalities was selected to..."
**STATISTICAL METHODS**	12	(a) Describe all statistical methods, including those used to control for confounding	**2.4. Statistical Analysis**<br><br>"All statistical analyses accounted for the complex..."
		(b) Describe any methods used to examine subgroups and interactions	**2.4. Statistical Analysis**<br><br>"A survey-weighted multivariable logistic regression model..."
		(c) Explain how missing data were addressed	**2.1. Data Source and Study Population**<br><br>"Participants were included if they aged at least..." (Complete case analysis implied by inclusion criteria)
		(d) If applicable, describe analytical methods taking account of sampling strategy	**2.4. Statistical Analysis**<br><br>"All statistical analyses accounted for the complex..."
		(e) Describe any sensitivity analyses	**4.1. Strengths and Limitations**

			"Finally, sensitivity analyses using alternative definitions..."
**RESULTS**			
**PARTICIPANTS**	13*	(a) Report numbers of individuals at each stage of study—eg numbers potentially eligible, examined for eligibility, confirmed eligible, included in the study, completing follow-up, and analysed	**3. Results**<br><br>"The weighted analytic sample represented approximately 60 million..."
		(b) Give reasons for non-participation at each stage	**2.1. Data Source and Study Population**<br><br>"Participants were included if they aged at least..."
**DESCRIPTIVE DATA**	14*	(a) Give characteristics of study participants (eg demographic, clinical, social) and information on exposures and potential confounders	**3. Results (Table 1)**<br><br>"As detailed in Table 1, the MUNW population..."
		(b) Indicate number of participants with missing data for each variable of interest	**2.1. Data Source and Study Population**<br><br>"...and had complete data for diabetes diagnosis..."
**OUTCOME DATA**	15*	Report numbers of outcome events or summary measures	**3. Results**<br><br>"The prevalence of undiagnosed diabetes in the MHNW..."
**MAIN RESULTS**	16	(a) Give unadjusted estimates and, if applicable, confounder-adjusted estimates and their precision (eg, 95% confidence interval). Make clear which confounders were adjusted for and why they were included	**3. Results (Table 2)**<br><br>"In the fully adjusted logistic regression model..."
		(b) Report category boundaries when continuous variables were categorized	**2.2. Definition of Metabolic Phenotypes**<br><br>"...elevated triglycerides (≥ 150 mg/dL), reduced..."

		(c) If relevant, consider translating estimates of relative risk into absolute risk for a meaningful time period	**N/A**
**OTHER ANALYSES**	17	Report other analyses done—eg analyses of subgroups and interactions, and sensitivity analyses	**3. Results**<br><br>"Non-Hispanic Asian adults exhibited a markedly elevated..."
**DISCUSSION**			
**KEY RESULTS**	18	Summarize key results with reference to study objectives	**4. Discussion**<br><br>"This study identifies a critical gap in diabetes..."
**LIMITATIONS**	19	Discuss limitations of the study, taking into account sources of potential bias or imprecision. Discuss both direction and magnitude of any potential bias	**4.1. Strengths and Limitations**<br><br>"However, the reliance on BMI as a primary selection..."
**INTERPRETATION**	20	Give a cautious overall interpretation of results considering objectives, limitations, multiplicity of analyses, results from similar studies, and other relevant evidence	**4. Discussion**<br><br>"Our results highlight that "normal weight" has different..."
**GENERALIZABILITY**	21	Discuss the generalizability (external validity) of the study results	**4. Discussion**<br><br>"...applied to the US population, this represents..."
**OTHER INFORMATION**			
**FUNDING**	22	Give the source of funding and the role of the funders for the present study and, if applicable, for the original study on which the present article is based	**Funding**<br><br>"This research received no external funding."
